# Genetic Mapping of Resistance to *Meloidogyne arenaria* in *Arachis stenosperma*: A New Source of Nematode Resistance for Peanut

**DOI:** 10.1534/g3.115.023044

**Published:** 2015-12-10

**Authors:** Soraya C. M. Leal-Bertioli, Márcio C. Moretzsohn, Philip A. Roberts, Carolina Ballén-Taborda, Tereza C. O. Borba, Paula A. Valdisser, Rosana P. Vianello, Ana Cláudia G Araújo, Patricia M. Guimarães, David J. Bertioli

**Affiliations:** * Embrapa Genetic Resources and Biotechnology, PqEB W5 Norte Final, Brasília, DF, 70770-917, Brazil; † Center for Applied Genetic Technologies, University of Georgia, Athens, Georgia 30602-6810; ‡ Department of Nematology, University of California, Riverside, California 92521; § Embrapa Rice and Beans, Rodovia GO-462, km 12 Zona Rural C.P. 179, Santo Antônio de Goiás, GO, 75375-000, Brazil; ** University of Brasília, Institute of Biological Sciences, Campus Darcy Ribeiro, Brasília, DF, 70910-900, Brazil

**Keywords:** *Arachis*, peanut, QTL, root-knot nematode resistance, marker-assisted selection, drought, yield, introgression

## Abstract

Root-knot nematodes (RKN; *Meloidogyne* sp.) are a major threat to crops in tropical and subtropical regions worldwide. The use of resistant crop varieties is the preferred method of control because nematicides are expensive, and hazardous to humans and the environment. Peanut (*Arachis hypogaea*) is infected by four species of RKN, the most damaging being *M. arenaria*, and commercial cultivars rely on a single source of resistance. In this study, we genetically characterize RKN resistance of the wild *Arachis* species *A. stenosperma* using a population of 93 recombinant inbred lines developed from a cross between *A. duranensis* and *A. stenosperma*. Four quantitative trait loci (QTL) located on linkage groups 02, 04, and 09 strongly influenced nematode root galling and egg production. Drought-related, domestication and agronomically relevant traits were also evaluated, revealing several QTL. Using the newly available *Arachis* genome sequence, easy-to-use KASP (kompetitive allele specific PCR) markers linked to the newly identified RKN resistance loci were developed and validated in a tetraploid context. Therefore, we consider that *A. stenosperma* has high potential as a new source of RKN resistance in peanut breeding programs.

Nematodes of the genus *Meloidogyne*, or root-knot nematodes (RKN) cause significant economic losses in agricultural crops worldwide. RKNs are sedentary obligate plant endoparasites, and, as a result of nematode feeding, large galls or “knots” are formed throughout the root system of infected plants. Severe infections reduce yields in numerous crops and can also affect consumer acceptance of many plants, especially vegetables. RKNs establish a complex biotrophic relationship with their hosts. Second-stage juveniles invade root tip cells, migrate through the root cortex, and, after electing suitable root cells, induce redifferentiation into specialized feeding cells. Feeding cells enlarge and are converted into multinucleate giant cells through synchronous nuclear divisions without cell division. Hyperplasia and hypertrophy of the surrounding cortical cells lead to the formation of the typical root gall, the primary visible symptom of infection. Plant nutrient and water uptake is substantially reduced by the resulting damage to the root system, and infested plants are therefore weak and low yielding (Caillaud *et al.* 2008). Management of nematodes typically includes use of chemicals, crop rotation, and use of resistant cultivars. Most chemical control agents against RKNs have been prohibited for environmental and health reasons (De Waele *et al.* 1989), and crop rotation is limited because of the wide range of hosts of RKN (Dong *et al.* 2007). Therefore, utilization of resistant cultivars is considered the best alternative for production in nematode-infested areas.

Peanut (*Arachis hypogaea* L.), an important oilseed and food crop worldwide, is affected by four RKN species, *M. hapla* Chitwood, *M. javanica* (Treub) Chitwood, *M. haplanaria* n. sp., and *M. arenaria* (Neal) Chitwood, with the latter being the most destructive (Carneiro *et al.* 2003; Eisenback *et al.* 2003). Cultivated peanut has only moderate levels of resistance to RKN (Holbrook and Stalker 2003), whereas wild relatives of peanut harbor much greater levels of resistance (Nelson *et al.* 1989; Holbrook and Noe 1990). Resistance to RKN has been introgressed into peanut from its wild relative *A. cardenasii* Krapov. & W.C. Greg. through the tetraploid (Simpson *et al.* 1993), and the hexaploid introgression pathways (Garcia *et al.* 1995; Stalker *et al.* 2002). In infested regions, the use of resistant cultivars that harbor resistance from this wild species, such as COAN, NemaTAM, Tifguard, Webb, Tifguard High O/L is essential for production and profitability (Simpson and Starr 2001; Simpson *et al.* 2003, 2013; Holbrook *et al.* 2008).

The resistance to RKN in these modern varieties is derived from a single chromosome segment from *A. cardenasii* (Burow *et al.* 2001; Nagy *et al.* 2010). However, since only a single source of resistance is used, there is a clear possibility that the resistance will be broken. New sources of resistances are very likely to be needed. Wild relatives of peanut are a rich source of alleles for resistance to biotic and abiotic stresses because they have been selected during evolution in a range of environments (Stalker and Moss 1987; Leal-Bertioli *et al.* 2012). In particular, *A. stenosperma* Krapov. & W. C. Greg has been shown to be highly resistant to *M. arenaria* (Proite *et al.* 2008; Leal-Bertioli *et al.* 2010).

*A. stenosperma* is also resistant to several fungal pathogens of peanut, including rust (*Puccinia arachidis* Speg.), late leaf spot (*Cercosporidium personatum* Berk. & M.A. Curtis), web blotch (*Phoma arachidicola* Marasas, Pauer & Boerema), and scab (*Sphaceloma arachidis* Bitanc. & Jenkins) (Leal-Bertioli *et al.* 2010; Michelotto *et al.* 2015). Furthermore, *A. stenosperma* is relatively conservative in terms of water use under limited availability, showing a higher soil moisture threshold for transpiration decline than the cultivated peanut (Leal-Bertioli *et al.* 2012, and unpublished data). To study the genetics of these potentially valuable traits we have previously developed and characterized a diploid mapping population from a cross of *A. duranensis* (the A-subgenome ancestor of cultivated peanut) and *A. stenosperma* (Moretzsohn *et al.* 2005; Leal-Bertioli *et al.* 2009; Shirasawa *et al.* 2013). To enable the introgression of its wild alleles into cultivated peanut we have developed *A. stenosperma*-derived induced allotetraploids that are sexually compatible with *A. hypogaea* (Leal-Bertioli *et al.* 2015c).

In this work, we identified genomic regions that control two main components of nematode infection: gall and egg production. Quantitative trait loci (QTL) were identified in positions distinct from the genetic location of the *A. cardenasii* chromosomal segment introgressed previously, indicating that the genes involved are different. QTL were also identified for drought-related, domestication, and agronomically important traits. KASP (kompetitive allele specific PCR) markers were designed for the genome regions that confer strongest nematode resistance and validated in tetraploid backgrounds. We envisage that these markers will be useful for marker-assisted selection in breeding programs.

## Materials and Methods

### Plant material

*Arachis* species seeds were obtained from the Brazilian *Arachis* germplasm collection, maintained at Embrapa Genetic Resources and Biotechnology (Brasília-DF, Brazil). *A. monticola* seeds were obtained from the United States Department of Agriculture collection (USDA; http://www.ars-grin.gov/). The parental accessions for the recombinant inbred lines (RILs) were two A-genome accessions that contrast for nematode resistance (Proite *et al.* 2008): *A. duranensis* Krapov. & W. C. Greg. K7988 and *A. stenosperma* Krapov. & W. C. Greg. V10309 (USDA PI666100), used as the female and male parents, respectively. The F_2_ population derived from this cross was used in the genetic studies described in Moretzsohn *et al.* 2005, Bertioli *et al.* 2009 and Leal-Bertioli *et al.* 2009. The F_6_ RIL population used for this study composed of 93 individuals was obtained by single seed descent from this F_2_ population. Previous genetic studies of this population are described in Shirasawa *et al.* (2013) and Bertioli *et al.* (2014).

### Phenotyping

#### Nematode resistance:

The parents of the population, *A. durane*nsis K7988 and *A stenosperma* V10309, and the wild resistant accession *A. cardenasii* GKP10017 (PI648354), were evaluated for resistance to four nematode species: *M. hapla*, *M. arenaria* race 1, *M. arenaria* race 2, *M. javanica* race 4 (Carneiro *et al.* 2003), and the peanut pod nematode *Ditylenchus africanus* Wendt, Swart, Vrain & Webster (Wendt *et al.* 1995). The susceptible peanut cultivar IAC-Tatu (*A. hypogaea* subsp. *fastigiata* var. *fastigiata*) was used as susceptible control. All *Meloidogyne* populations were maintained on the susceptible tomato variety ‘Santa Cruz’ at Embrapa Genetic Resources and Biotechnology. *D. africanu*s was obtained from South Africa (De Waele *et al.* 1989), and multiplied in alfalfa plants *in vitro*. All assays were performed in a greenhouse under quarantine conditions. Plants (10- to 12-wk-old) were inoculated with 5000–10,000 eggs; 10 wk after inoculation, eggs were extracted from roots using 0.5% NaOCl (Hussey and Barker 1973), stained with acid fuchsin and counted using a Peters slide under the microscope. For *D. africanus*, nematodes were extracted from whole plants 35 d after inoculation. The nematode reproductive factor (RF) was calculated as RF = Pf/Pi (Oostenbrink 1966), where Pf = final nematode population, and Pi = initial nematode population. Average reproduction factors, log (*x* + 1) transformed, were compared by the Tukey test with significance at the 5% probability level. Treatments with RF < 1.00 were considered resistant to the nematode species and, those with RF > 1.0 as susceptible (Oostenbrink 1966).

Eighty-two lines of the recombinant inbred F_6_ population (*A. durane*nsis K7988 × *A. stenosperma* V10309), the parents and controls were evaluated for resistance to *M. arenaria* race 1. Bioassays were performed essentially as described in Morgante *et al.* (2013). Briefly, 4-wk-old plantlets were inoculated with 50,000 eggs of *M. arenaria* extracted from tomato cv. UC82 plants. Five replicate plants of each genotype were tested; the five sets of replicates were arranged on greenhouse benches in a randomized complete block design. Bioassays were performed in each of two years (2011 and 2013). The peanut cultivar Florunner (Norden *et al.* 1969) was used as a susceptible control. Temperature in the greenhouse was maintained between 28° and 35° in the day, and 24° at night. Root systems were washed free of soil and scored for phenotype 9 wk (experiment I—2011) or 11 wk (experiment II—2013) after inoculation. A 0–10 root-gall rating scale (Bridge and Page 1980) was used to evaluate resistance reaction to nematodes [root galling index (GI)] (Wang *et al.* 2012). Nematode reproduction was evaluated as another phenotypic component of resistance. Eggs were extracted in NaOCl from weighed root systems and counted to provide numbers of eggs per gram of root (EGR).

#### Agronomic, domestication, and drought-related traits:

Plants were grown in long trays (1 m × 30 cm × 30 cm), with enough space for lateral branch trailing and seed set. Branches were regularly trailed back to the pots to ensure that pegs would get to the soil. Between 40 and 60 d after planting, height of main stem (MSH) and lateral branches were counted (NLB), and measured (LBL). At harvest (about 120 d after planting), peg length (PL) was measured on six pods. Harvested seeds were counted (SN), dried at 20° at 15% RH for 15 d, and then weighed. Pod isthmus was measured (Pod_constr). Plants were oven-dried for 96 hr at 80°. Aerial parts and roots were weighed separately (ADW, RDW), and the total weight, including that of seeds was added, and comprised total biomass (TB). The weight of 10 seeds (10-SW), randomly selected, was used for QTL analyses. Evaluations were conducted in each of 2 years. Pollen viability (PV) was estimated by the staining method with acetic carmine (Linsley and Cazier 1963). For each genotype, 1000 pollen grains were analyzed from oblong anthers as follows: 100 pollen grains per anther, two anthers per flower, and five flowers per plant.

Drought-related traits SPAD chlorophyll meter reading (SCMR), and specific leaf area (SLA), were evaluated on the first expanded leaves of four lateral branches of each F_6_ plant and parents, as described in Leal-Bertioli *et al.* (2012). All SCMR and SLA evaluations were performed in the morning, at three stages: 40, 60, and 120 d after germination. Transpiration per total leaf area (TR/LA, proxy for stomatal conductance) was evaluated on the parents. Transpiration was measured gravimetrically on well-watered plants over three subsequent days. TR/LA was expressed as g/cm.

### Statistical analysis

Phenotypic data were analyzed using the Statistical package R (R team). Data normality was tested using the Shapiro test. Tukey HSD test (normally distributed data) and Kruskal-Wallis one-way analysis of variance by ranks (non-normally distributed data) were used for comparison of averages at *P* = 5%. For QTL identification, non-normal data were transformed to Log10 (*x* + 1).

### Marker development and genotyping

Total genomic DNA extraction and quantification were performed essentially as described in Leal-Bertioli *et al.* (2015b). Single nucleotide polymorphisms (SNPs) were identified using transcriptome of roots of young seedlings and developing seeds of *A. duranensis* PI 475887, and *A. duranensis* Grif 15036 (Nagy *et al.* 2012). SNPs were also identified between *A. duranensis* K7988 and *A. stenosperma* V10309 ESTs (Guimarães *et al.* 2012). SNP genotyping was performed using the GoldenGate Illumina array described by Nagy *et al.* (2012), and calling of genotypes was using GenomeStudio 2011.1. Scores to each data point were assigned using the software GenCall. The GenCall score is a value between zero and one, and is primarily designed to filter out failed genotypes, DNAs, and/or loci (Oliphant *et al.* 2002). Scores less than 0.2 usually indicate failed assays, and more than 0.7 usually report high-quality genotypes. All markers used for map construction are described in Supporting Information, File S1.

### Genetic mapping and QTL analyses

Two linkage maps for this same RIL population have been previously constructed (Bertioli *et al.* 2014; Shirasawa *et al.* 2013). We used all genotyped markers of these two studies plus SNP markers genotyped in the present work to construct a saturated map using JoinMap 4.0 (Van Ooijen 2006). Based on this map, genomic regions with no recombination or identical markers were identified, and all loci but one were removed from these regions. Remaining loci were used to construct a framework map using Mapmaker Macintosh 2.0 (Lander *et al.* 1987; Lincoln *et al.* 1992). A χ^2^ test was performed to test the null hypothesis of 1:1 segregation on all scored markers. A minimum LOD score of 9.0 and maximum recombination fraction of 0.35 were set as thresholds for linkage groups (LG) determination with the “group” command. The most likely marker order within each LG was estimated by the matrix correlation method using the “first order” command. Marker orders were confirmed by comparing the log-likelihood of the possible orders by permuting all adjacent triple orders (“ripple” command). After establishment of the group orders, the LOD score was set to 3.0 in order to include additional markers in the groups. The “try” command was then used to determine the exact position of the new markers within each group. The new marker orders were again confirmed with the “ripple” command. Recombination fractions were converted into map distances in centimorgans (cM) using the Kosambi’s mapping function (Lander *et al.* 1987; Lincoln *et al.* 1992).

This newly developed framework map was used for QTL analysis. Phenotyping data included: components of resistance to *M. arenaria* race 1 and drought-related, domestication and agronomic traits (File S1). Traits evaluated in different trials or years were analyzed separately. The normality of data distribution was evaluated by skewness and kurtosis values using WinQTL Cartographer, version 2.5 (Wang *et al.* 2006). QTL were mapped by using the composite interval mapping (CIM) method, proposed by Zeng (1993, 1994) also using WinQTL Cartographer. Some of the data sets were non-normally distributed and were log transformed. CIM analysis used the Standard Model (Model 6), scanning the genetic map, and estimating the likelihood of a QTL and its corresponding effects at every 1 cM, while using eight significant marker cofactors to adjust the phenotypic effects associated with other positions in the genetic map. A window size of 10 cM was used, and therefore cofactors within 10 cM on either side of the QTL test site were not included in the QTL model. Thresholds were determined for each trait by permutation tests (Churchill and Doerge 1994; Doerge and Churchill 1996), using 1,000 permutations and a significance level of 0.05. Graphic presentation of the LGs and the significant QTL was drawn with MapChart, version 2.1 (Voorrips 2002).

The effect of markers linked to QTL contributing to nematode resistance was analyzed individually and cumulatively. For the first analyses, the phenotypic average of the RILs with each of the positive alleles (presence of the marker closest linked to the QTL) was calculated and compared with the average of the RILs without the positive alleles. To analyze the cumulative effect of the alleles, phenotypic averages of the RILs with any combination of 0, 2, 4 or 6 positive alleles were compared. Class-specific means of GI and EGR and standard errors were calculated for each genotypic class.

### KASP marker development and validation on tetraploid backgrounds

The longer-term aim of this research is the introgression of the *A. stenosperma* chromosomal segments that confer nematode resistance into cultivated peanut by marker-assisted backcrossing. For this, it is necessary that the markers function within a tetraploid genetic context. We tested a strategy that uses the genome sequence of *A. duranensis* V14167 (http://www.peanutbase.org). In principle this strategy allows the development of markers to directed chromosomal regions. Also because of the inclusion of *A. hypogaea* controls in the marker tests, the results of the test would give a measure of how well the genome sequence of *A. duranensis* V14167 serves as a proxy for the A-subgenome of *A. hypogaea*.

#### SNP discovery:

SNPs were discovered by aligning sequences from the nematode resistant *A. stenosperma* V10309 with the reference genome of *A. duranensis* using the Bowtie2 pipeline (Langmead & Salzberg 2012) by tagging the specific regions where the main QTL for nematode resistance were identified on pseudomolecules Adur.A02, Adur.A04 and Adur.A09, using default parameters. SNPs were called using SAMtools (Li *et al.*, 2009).

#### Primer design and test:

Allele-specific forward primers and a common reverse primer were designed for use in KASP (Kompetitive Allele Specific PCR) assays (LGC Genomics Ltd. Hoddesdon, U.K.), using BatchPrimer3 (http://probes.pw.usda.gov/batchprimer3/) with the “Allele specific primers and allele flanking primers” option. The parameter used were 60–120 bp in size, Tm between 58–60° and GC content between 30 and 80%. The alternative alleles were marked with 6-FAM and reference alleles (*A. duranensis* V14167, http://www.peanutbase.org) with VIC. For each SNP, two allele-specific forward primers, and one common reverse primer were designed, essentially as described in (Leal-Bertioli *et al.* 2015b). All KASP primers are listed on Table 1.

**Table 1:** Information about KASP assays, including primer name (with linkage group, position on *A. duranensis* pesudomolecule, orientation, and dye), primer sequence and type, melting temperature, GC content, and SNP type amplification pattern

Primer Name (LG, Position_Orientation_Dye)*^a^*	Sequence	Type	Tm	GC%	SNP	Amplification pattern*^b^*
Nem_Aradu.A02_76738828_Fwd_	CAACTAAGCAACAGGAAAGACG	AF	58.93	47.62		(As = BatSten = GregSten) ≠ Ad ≠ (Ah = Am)*
Nem_Aradu.A02_76738828_Rev_FAM	GAAGGTGACCAAGTTCATGCTGATCATTGTTGCCGAATCTC	AS	58.09	45	A	
Nem_Aradu.A02_76738828_Rev_VIC	GAAGGTCGGAGTCAACGGATTGATCATTGTTGCCGAATCTT	AS	57.61	40	G	
Nem_Aradu.A02_83608917_Fwd_	TTTGTGGCTGCAATAACTTCA	AF	59.36	38.1		Ad ≠ (As = BatSten = GregSten = Ah = Am)
Nem_Aradu.A02_83608917_Rev_VIC	GAAGGTCGGAGTCAACGGATTCATGACATTGTAAGTGGCAAAAAC	AS	60.66	37.5	G	
Nem_Aradu.A02_83608917_Rev_FAM	GAAGGTGACCAAGTTCATGCTCATGACATTGTAAGTGGCAAAAAT	AS	60.17	33.33	A	
Nem_Aradu.A02_84440546_Rev_	GCGATTAATACATTCAACAACCA	AF	58.93	34.78		(As = BatSten = GregSten) ≠ (Ad = Ah = Am)*
Nem_Aradu.A02_84440546_Fwd_FAM	GAAGGTGACCAAGTTCATGCTGCTCTCCTTCTTGGTGGTTTG	AS	61.17	52.38	A	
Nem_Aradu.A02_84440546_Fwd_VIC	GAAGGTCGGAGTCAACGGATTGCTCTCCTTCTTGGTGGTTTA	AS	58.45	47.62	G	
Nem_Aradu.A02_84440594_Rev_	GGAAGCGGATTCCACTCA	AF	59.72	55.56		(As = BatSten = GregSten) ≠ Ad ≠ (Ah = Am)*
Nem_Aradu.A02_84440594_Fwd_FAM	GAAGGTGACCAAGTTCATGCTGAAGTGTGTCATAATCTCCAAAGTG	AS	59.12	40	A	
Nem_Aradu.A02_84440594_Fwd_VIC	GAAGGTCGGAGTCAACGGATTCTGAAGTGTGTCATAATCTCCAAAGTA	AS	60	37.04	G	
DS_c1614_886_A02_88903581_Rev_	AGCTGAGGAGAACCCCTTTT	AF	59.32	50		Ad ≠ (As = BatSten = GregSten = Ah = Am)
DS_c1614_886_A02_88903581_Fwd_FAM	GAAGGTGACCAAGTTCATGCTCAGATACAGTGACAGATATGAATGGTG	AS	61.02	40.74	G	
DS_c1614_886_A02_88903581_Fwd_VIC	GAAGGTCGGAGTCAACGGATTTCAGATACAGTGACAGATATGAATGGTA	AS	60.2	35.71	A	
TOG894171_695_A02_92486807_Rev_	CTTCTGTTGGGGTGTTGGAT	AF	59.82	50		(As = BatSten = GregSten) ≠ (Ad = Ah = Am)*
TOG894171_695_A02_92486807_Fwd_VIC	GAAGGTCGGAGTCAACGGATTYATTAATCAGGCAATAGCAACG	AS	59.65	36.36	G	
TOG894171_695_A02_92486807_Fwd_FAM	GAAGGTGACCAAGTTCATGCTCTAYATTAATCAGGCAATAGCAACA	AS	59.62	32	A	
Nem_Aradu.A02_92631394_Fwd_	AAGAAATTGGGCGTTTTCAG	AF	68	118		(As = Ah = Am) ≠ Ad ≠ (BatSten = GregSten)*
Nem_Aradu.A02_92631394_Rev_FAM	GAAGGTGACCAAGTTCATGCTATCCCCATATCTAGTGTCTTCTGC	AS	59.89	45.83	A	
Nem_Aradu.A02_92631394_Rev_VIC	GAAGGTCGGAGTCAACGGATTCAATCCCCATATCTAGTGTCTTCTGT	AS	60.94	42.31	G	
Nem_Aradu.A04_109789467_Rev_	CCAAAGCTCTTTTCCAGGTT	AF	58.44	45		(As) ≠ (BatSten = GregSten) ≠ (Ad = Ah = Am)*
Nem_Aradu.A04_109789467_Fwd_FAM	GAAGGTGACCAAGTTCATGCTCAATAGAAACAGCAAAGCAATGG	AS	60.98	39.13	A	
Nem_Aradu.A04_109789467_Fwd_VIC	GAAGGTCGGAGTCAACGGATTCAATAGAAACAGCAAAGCAATGA	AS	59.44	34.78	G	
TOG906490_74_A04_106874754_Fwd_	TTCATTCCATAAGCCCAACC	AF	59.76	45		(As = BatSten = GregSten) ≠ (Ad = Ah = Am)*
TOG906490_74_A04_106874754_Rev_VIC	GAAGGTCGGAGTCAACGGATTAACTTTTCGAATCCTCATATTGCT	AS	59.57	33.33	A	
TOG906490_74_A04_106874754_Rev_FAM	GAAGGTGACCAAGTTCATGCTTTTTCGAATCCTCATATTGCG	AS	60.05	38.1	C	
TOG937303_589_A04_108564975_Rev_	CCATCACAAAAGAACAAAACAAC	AF	58.57	34.78		ND
TOG937303_589_A04_108564975_Fwd_FAM	GAAGGTGACCAAGTTCATGCTAATTACTCGTTGGAGTAGTTGATGG	AS	59.85	40	G	
TOG937303_589_A04_108564975_Fwd_VIC	GAAGGTCGGAGTCAACGGATTGAATTACTCGTTGGAGTAGTTGATGA	AS	59.96	38.46	A	
Nem_Aradu.A04_113373632_Rev_	TCCTCATCATCATCTTTCTCCA	AF	59.63	40.91		ND
Nem_Aradu.A04_113373632_Fwd_FAM	GAAGGTGACCAAGTTCATGCTAGGTTGGTCAAGGGTTTCAG	AS	59.04	50	A	
Nem_Aradu.A04_113373632_Fwd_VIC	GAAGGTCGGAGTCAACGGATTAGGTTGGTCAAGGGTTTCAA	AS	59.42	45	G	
TOG896942_133_A09_114770700_Fwd_	AAAGAAAGGGCTCCCTAATTTC	AF	59.16	40.91		(As = BatSten = GregSten) ≠ (Ad = Ah = Am)*
TOG896942_133_A09_114770700_Rev_FAM	GAAGGTGACCAAGTTCATGCTGGGCACAAAAATTCGCTACA	AS	61	45	T	
TOG896942_133_A09_114770700_Rev_VIC	GAAGGTCGGAGTCAACGGATTGGCACAAAAATTCGCTACG	AS	59.32	47.37	C	
Nem_Aradu.A04_114769893_Fwd_	TCAAGTCGTGTGTTCTCTACACC	AF	59.32	47.83		(As = BatSten = GregSten = Ad = Ah = Am)
Nem_Aradu.A04_114769893_Rev_FAM	GAAGGTGACCAAGTTCATGCTTCTTGTGACATGAGCTACAACTTCT	AS	59.53	40	C	
Nem_Aradu.A04_114769893_Rev_VIC	GAAGGTCGGAGTCAACGGATTTTGTGACATGAGCTACAACTTCG	AS	60.35	43.48	A	
Nem_Aradu.A04_115457181_Rev_	TGTGGACAGATGGAAAACACA	AF	59.99	42.86		(As = GregSten) ≠ (BatSten = Ad = Ah = Am)*
Nem_Aradu.A04_115457181_Fwd_VIC	GAAGGTCGGAGTCAACGGATTTTCGGCGTTGGACTGTG	AS	60.4	58.82	G	
Nem_Aradu.A04_115457181_Fwd_FAM	GAAGGTGACCAAGTTCATGCTCTTCGGCGTTGGACTGTA	AS	58.35	55.56	A	
Nem_Aradu.A04_117955004_Fwd_	TCACGGTCCATGTATTCAGC	AF	59.53	50		(As = GregSten = BatSten = Am) ≠ (Ad = Ah)*
Nem_Aradu.A04_117955004_Rev_VIC	GAAGGTCGGAGTCAACGGATTCGTTAGCAGTTGGACAAACAAAC	AS	59.76	50	G	
Nem_Aradu.A04_117955004_Rev_FAM	GAAGGTGACCAAGTTCATGCTCGTTAGCAGTTGGACAAACAAAT	AS	60.95	43.48	A	
Nem_Aradu.A04_121132127_Rev_	AGATTTTCTGGGCCCATTTT	AF	59.78	40		(As = BatSten = GregSten) ≠ (Ad = Ah = Am)*
Nem_Aradu.A04_121132127_Fwd_VIC	GAAGGTCGGAGTCAACGGATTGCCAAGCAAAGTAATGCCG	AS	61.67	52.63	G	
Nem_Aradu.A04_121132127_Fwd_FAM	GAAGGTGACCAAGTTCATGCTGCCAAGCAAAGTAATGCCA	AS	59.82	47.37	A	
Nem_Aradu.A04_121183243_Rev_	AAGGTTGGGAATGTCAAGGA	AF	59.38	45		(As = BatSten = GregSten = Ad = Ah = Am)
Nem_Aradu.A04_121183243_Fwd_FAM	GAAGGTGACCAAGTTCATGCTAACTGGTAGGTTTGGAAATAATCG	AS	59.7	37.5	C	
Nem_Aradu.A04_121183243_Fwd_VIC	GAAGGTCGGAGTCAACGGATTAAACTGGTAGGTTTGGAAATAATCC	AS	59.91	36	G	
Nem_Aradu.A09_112396428_Fwd_	TATGATTGGCCCCCTAAATG	AF	59.62	45		(As = BatSten = GregSten) ≠ Ad ≠ (Ah = Am)*
Nem_Aradu.A09_112396428_Rev_FAM	GAAGGTGACCAAGTTCATGCTAGCCCCCTCTTCTAAAACAAC	AS	58.77	47.62	A	
Nem_Aradu.A09_112396428_Rev_VIC	GAAGGTCGGAGTCAACGGATTCAGCCCCCTCTTCTAAAACAAT	AS	60.81	45.45	G	
Nem_Aradu.A09_112396635_Rev_	CCTGGCTTCATGTTTGATGA	AF	59.65	45		Ad ≠ (As = BatSten = GregSten = Ah = Am)
Nem_Aradu.A09_112396635_Fwd_VIC	GAAGGTCGGAGTCAACGGATTAATGTTACAAAAGGATCCCCAG	AS	59.24	40.91	G	
Nem_Aradu.A09_112396635_Fwd_FAM	GAAGGTGACCAAGTTCATGCTAATGTTACAAAAGGATCCCCAA	AS	59.59	36.36	A	
Nem_Aradu.A09_112399976_Rev_	TGACGAGAAGGGGAAAGAAA	AF	59.78	45		(As = BatSten = GregSten = Ad = Ah = Am)
Nem_Aradu.A09_112399976_Fwd_FAM	GAAGGTGACCAAGTTCATGCTAATCCTATTACTAAATCGCTGCTTTT	AS	59.27	30.77	C	
Nem_Aradu.A09_112399976_Fwd_VIC	GAAGGTCGGAGTCAACGGATTAATCCTATTACTAAATCGCTGCTTTC	AS	59.65	34.62	G	
Nem_Aradu.A09_112901114_Rev_	CTCCCCAATTTCTCAGCAAG	AF	59.81	50		(As) ≠ (BatSten = GregSten) ≠ (Ad = Ah = Am)*
Nem_Aradu.A09_112901114_Fwd_FAM	GAAGGTGACCAAGTTCATGCTAGGTGTTGACAGAATTACAACCG	AS	60.32	43.48	A	
Nem_Aradu.A09_112901114_Fwd_VIC	GAAGGTCGGAGTCAACGGATTGAGGTGTTGACAGAATTACAACCA	AS	60.32	41.67	G	
Nem_Aradu.A09_114001128_Rev_	TTAAAGCCCCTGCTTTTTCA	AF	59.83	40		(As = BatSten = GregSten) ≠ (Ad = Ah = Am)*
Nem_Aradu.A09_114001128_Fwd_FAM	GAAGGTGACCAAGTTCATGCTATGAGGGAACAACCAGCACTA	AS	59.61	47.62	C	
Nem_Aradu.A09_114001128_Fwd_VIC	GAAGGTCGGAGTCAACGGATTTGAGGGAACAACCAGCACTC	AS	61.26	55	A	
DS_c14276_456_A09_115161052_Rev_	AGGAGTCATGGGATGGAATG	AF	59.74	50		(As = Ad = GregSten) ≠ (BatSten = Ah = Am)*
DS_c14276_456_A09_115161052_Fwd_VIC	GAAGGTCGGAGTCAACGGATTTTTGGAAACATCAGCAAAGGA	AS	60.6	38.1	A	
DS_c14276_456_A09_115161052_Fwd_FAM	GAAGGTGACCAAGTTCATGCTTGGAAACATCAGCAAAGGC	AS	59.79	47.37	C	
TOG896078_413_A09_116503861_Rev_	GTGGAAGAAATAGCAAAATGGA	AF	58.25	36.36		(As = BatSten = GregSten) ≠ (Ad = Ah = Am)*
TOG896078_413_A09_116503861_Fwd_VIC	GAAGGTCGGAGTCAACGGATTAAGGAGTTATGGAGATGGTAAGTTTT	AS	59.02	34.62	T	
TOG896078_413_A09_116503861_Fwd_FAM	GAAGGTGACCAAGTTCATGCTAAGGAGTTATGGAGATGGTAAGTTTC	AS	59.41	38.46	C	
TOG903757_1119_A09_116533871_Rev_	CCCAAGAAGCAGGGTACTTT	AF	58.32	50		(As = BatSten = GregSten = Ad = Ah = Am)
TOG903757_1119_A09_116533871_Fwd_VIC	GAAGGTCGGAGTCAACGGATTACTTGATTTGATATGAGATTTCCTG	AS	57.83	32	G	
TOG903757_1119_A09_116533871_Fwd_FAM	GAAGGTGACCAAGTTCATGCTCACTTGATTTGATATGAGATTTCCTC	AS	59.45	34.62	C	

LG, Linkage group; TM, melting temperature; GC%, GC content; AF, Allele Flanking; As, *Arachis stenosperma*; BatSten, (*Arachis batizocoi* × *A. stenosperma*)^4x^, GregSten = (*Arachis gregoryi* × *A. stenosperma*)^4x^; Ad, *Arachis duranensis*; Ah, *Arachis hypogaea*; Am, *Arachis monticola*; AS, Allele specific; ND, Not defined/assay did not work.

a Dye: Reference (*A. duranensis*) alleles are coupled with VIC and alternative (*A. stenosperma*) alleles are coupled with FAM.

b Asterisk indicate assays that distinguish *A. stenosperma*-derived allotetraploids from *A. hypogaea*.

KASP assays were performed with the following genotypes: the diploids *A. duranensis* V14167 (A-reference genome), *A. stenosperma* V10309 (A-genome), the wild allotetraploid *A. monticola* accessions Pl219824 and Pl405933, the induced allotetraploids (*A. batizocoi* K9484 × *A. stenosperma* V10309)^4x^ (here called BatSten) and (*A. gregoryi* V6389 × *A. stenosperma* V10309)^4x^ (here called GregSten), and six *A. hypogaea* cultivars (Runner IAC-886, Tifrunner, Tifguard, GA-06G, NC3033, IAC69007). Reactions consisted of 2 μl of KASP 2X reaction mix, 0.055 μl of assay primer mix (12 mM of each allele-specific primer, and 30 mM of common primer), and 20 ng of genomic DNA, in a 4 µl volume. A C1000 Thermal Cycler (Bio-Rad) was used with the following cycling conditions: 94° for 15 min, nine cycles of 94° for 20 sec, touchdown starting at 65° for 60 sec (decreasing 0.8° per cycle), 29 cycles of 94° for 20 sec, and 57° for 60 sec. In order to improve the results, a second KASP program was run as following: nine cycles of 94° for 20 sec and 57° for 60 sec. Fluorescence was read by a LightCycler 480 Instrument II (Roche Life Science), and analyzed using the LightCycler 480 software (V.1.5.1). Three technical replicates were performed for each KASP assay.

In order to test correlation of KASP markers with nematode resistance, 20 of the most contrasting lines were selected to be assayed with the 15 KASP primers that successfully distinguished the synthetic allotetraploids from cultivated peanut.

## Results

### Nematode screening

**Figure 1:**
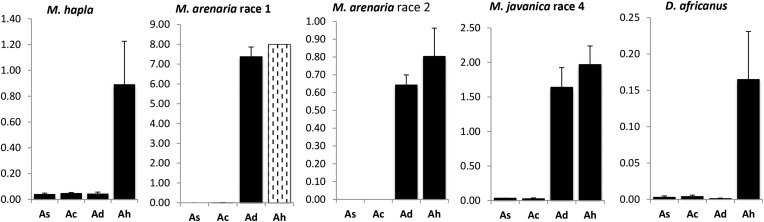
Reproductive factor of the nematodes *Meloidogyne hapla*, *M. arenaria* race 1, *M. arenaria* race 2, *M. javanica* race 4 and *Ditylenchus africanus*, observed in *A. stenosperma* (As), *A. cardenasii* (Ac), *A. duranensis* (Ad), and *A. hypogaea* cv. IAC Tatu (Ah). White bar with dashed vertical lines represents minimum expected RF of peanut plants that, due to heavy infestation, died before the end of the experiment.

*A. stenosperma* and *A. cardenasii* were resistant to all nematode species tested, hosting no gall or egg production (Figure 1). *A. duranensis* was resistant to *M. hapla* and *D. africanus*, and comparable to cultivated peanut in susceptibility to *M. javanica*, and *M. arenaria* race 2 (Tukey HSD, *P* = 0.05). To *M. arenaria* race 1, *A. duranensis* was partially resistant (Tukey HSD, *P* = 0.05, Figure 1). Resistance was evaluated as reproductive factor (RF). The peanut cultivar Tatu, used as positive control, was susceptible to all nematode species tested, and low RF was observed in the bioassays against *M. hapla* and *M. arenaria* race 2 and *D. africanus*. This was because, in all these very susceptible plants, the root system was severely damaged and fragmented, not sustaining large quantities of nematodes. All *A. hypogaea* plants inoculated with *M. arenaria* race 1 died before the end of the experiment (Figure 1).

### Population phenotyping

The F_6_ RIL population used here was produced by single seed descent from a cross of *A. duranensis* × *A. stenosperma*. Individuals show varying degrees of fertility. Pollen viability of segregating individuals ranged from 80% to 99.3%, reflecting the genetic distance of the parents. The population showed large variability for all traits tested.

#### Nematode resistance:

**Figure 2:**
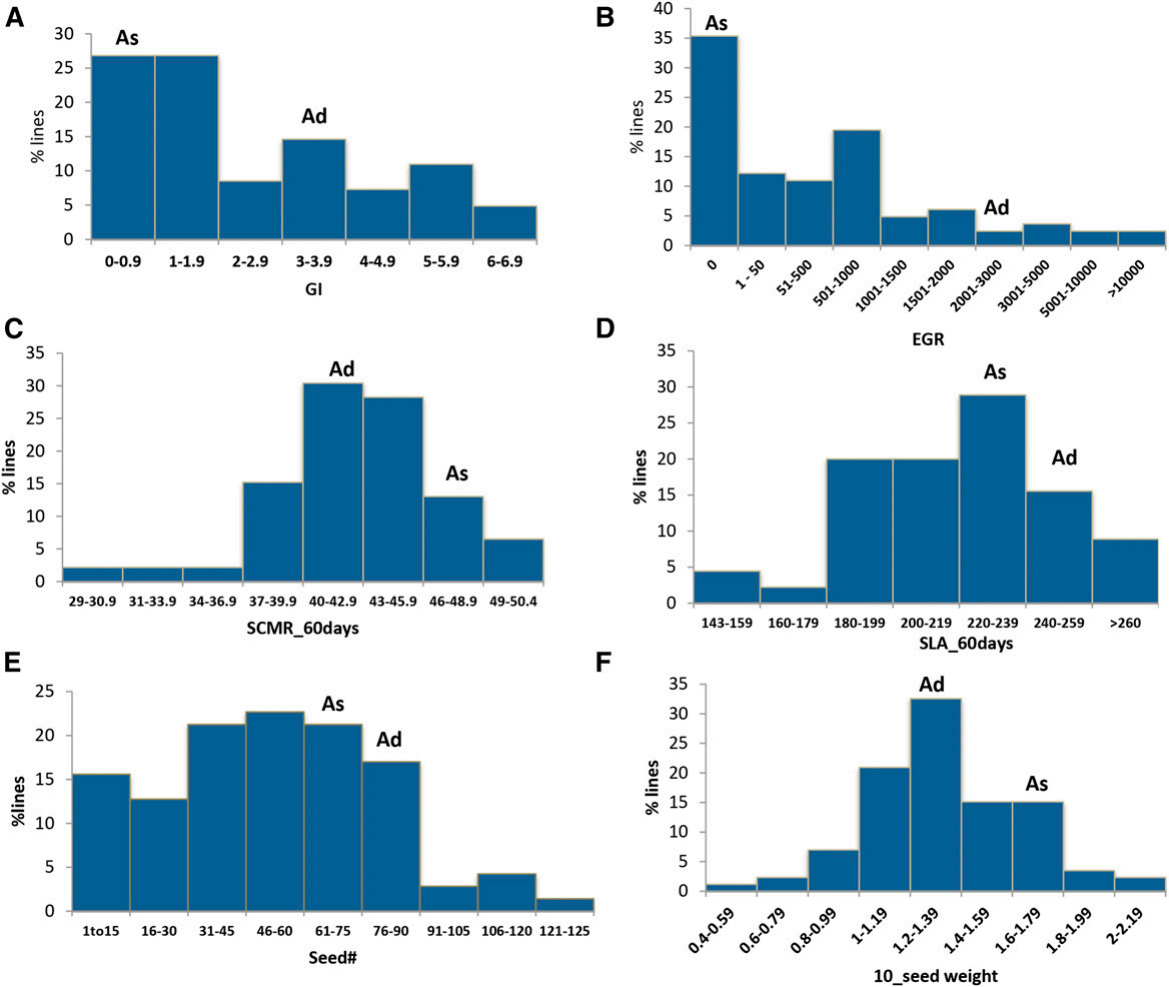
Frequency distribution of resistance to *Meloidogyne arenaria* race 1 (A–B), drought-related traits (C–D), and yield traits (E–F) in recombinant inbred lines (F_6_) derived from a cross of *A. duranensis* K7988 (Ad) with *A. stenosperma* V10309 (As). The means of the parents are significantly different (*P* < 0.05).

Evaluation was performed in the greenhouse for 2 years (2011 and 2013). Different severity levels were observed between experiments, with lower egg production levels in the second than in the first bioassay experiment. This difference might have been due to different environmental conditions, and also different nematode inoculum viability. All data were used for QTL identification but only the egg production data obtained from experiment I was used for statistical analyses, and evaluation of allele effects. Frequency distribution based on the pooled data for GI and EGR was strongly biased toward resistance. Some transgressive segregation was observed. For both GI datasets, most individuals had midparent values (54), only four were as, or more, resistant than *A. stenosperma* V10309 (0.3 ± 0.11) and 24 were more susceptible than *A. duranensis* K7988 (3.6 ± 0.86) (*P* < 0.05). For EGR, however, 31 individuals were more resistant than V10309 (10.16 ± 9.09), 42 had midparent values and only nine were more susceptible than K7988 (2188.89 ± 730.54) (*P* < 0.05). (Figure 2, A and B, and File S1). This showed that several lines that had high levels of root-galling supported only low levels of egg production. The susceptible peanut cultivar, Florunner had low infection rates. Wild species and segregating individuals showed smaller galls than Florunner. Pearson correlation between GI and EGR in the RIL population was significant [*r*^2^ (82) = 0.47, *P* = 0.01], but low enough to indicate that different genes might control the GI and nematode reproduction responses (Wang *et al.* 2012).

#### Drought-related traits:

The two parents showed little variation for the drought-related traits analyzed, with values differing numerically, but not statistically significantly (Kruskal-Wallis, *P* < 0.05). *A. stenosperma*: TR/LA = 0.245 ± 0.036 g/cm; SCMR (60) = 46.03 ± 2.08; SLA (60) = 209.58 ± 5.70 g/cm. *A. duranensis:* TR/LA = 0.323 ± 0.041 g/cm; SCMR (60) = 42.8 ± 1.72; SLA (60) = 239.09 ± 13.58 g/cm. The population showed large transgressive segregation for SCMR and SLA (Figure 2, C and D).

#### Agronomic and domestication traits:

Phenotypic evaluations were performed at different generations (F_5_ and F_6_) and places. Values were normally distributed for most traits for most years. With the exception of NLB and RRA, the means of the parents for all traits evaluated were significantly different (*P* < 0.05). *A. stenosperma* produced fewer but heavier seeds than *A. duranensis*. Comparison of the means of the parents and the segregating genotypes reveals that, for all traits, there was transgressive segregation in the progenies (Figure 2, E and F). This is particularly interesting for seed characteristics; for instance, 11 individuals outperformed both parents in seed production and five in seed weight.

### Construction of improved genetic map

**Figure 3:**
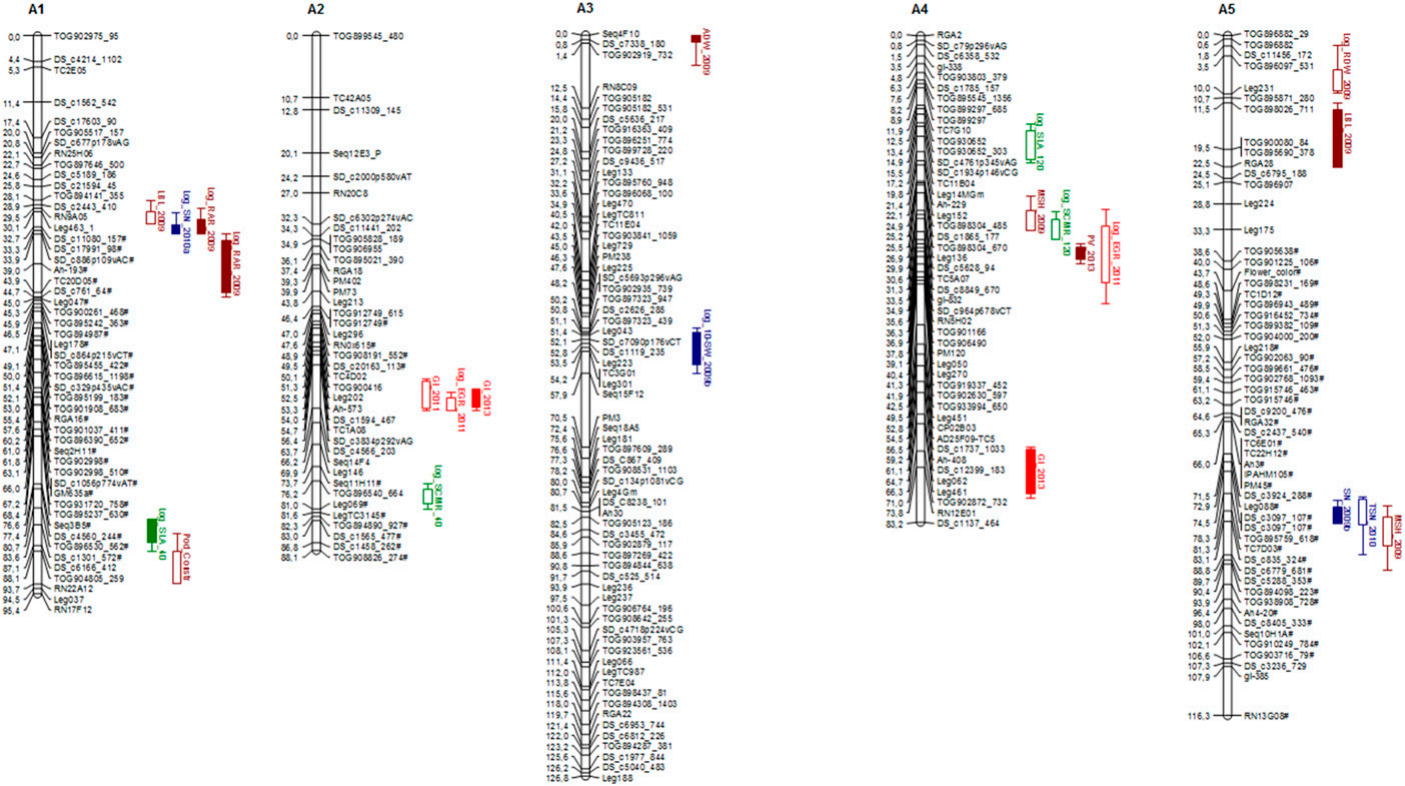

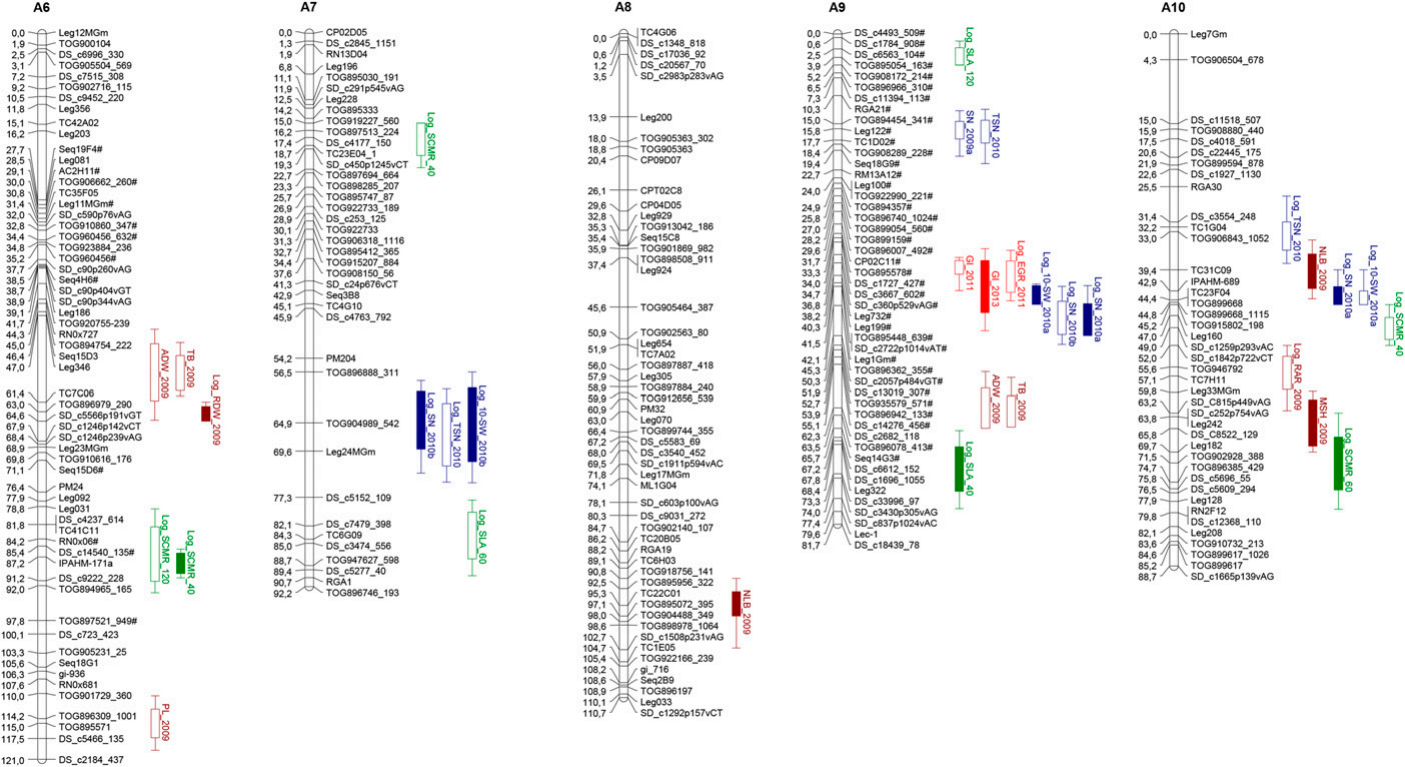
A genetic linkage map of the A-genome of *Arachis* obtained through the analysis of 90 F_6_ plants, generated from a cross between *A. duranensis* K7988 and *A. stenosperma* V10309. Numbers on the left of each group are Kosambi map distances (cM). QTL are indicated as colored bars running alongside linkage groups. Colors/textures are according to categories: red, nematode resistance; green, drought-related traits; blue, productivity; and brown, domestication and other agronomic traits. Distorted markers at *P* < 0.05 were identified by #.

Initially, a total of 1404 polymorphic markers were used for linkage map construction using JoinMap. With LOD scores ranging from 7 to 20, 1108 marker loci were mapped into 10 LGs, with a total distance of 490.4 cM (data not shown). These markers included 528 SSR, 511 SNP, 56 anchor, and 13 RGAs (resistance gene analog) markers. This map showed many genomic regions saturated of cosegregating markers. After removal of all but one of each set of cosegregating markers, and including the 296 (1404–1108) markers that did not map with JoinMap, a framework map was then constructed using Mapmaker. Using a minimum LOD score of 9.0 and a maximum recombination fraction of 0.35, 502 markers mapped onto 10 LGs, spanning a total map distance of 1004.1 cM. These markers included 316 SNPs, 96 SSRs, 72 anchor, 17 RGAs, and one morphological (flower color) marker. LGs were numbered according to the F_2_ reference map (Moretzsohn *et al.* 2005). LGs ranged from 81.7 cM (with 48 markers) to 126.8 cM (68 markers), with an average distance of 2.0 cM between adjacent markers. A total of 269 (53.6%) out of the 502 mapped markers deviated from the expected 1:1 ratio at *P* < 0.05 level. Of these, 165 markers were skewed toward *A. duranensis* and 104 markers toward *A. stenosperma*. LGs 1, 4, 6, 7, 8, and the upper portion of LG 2 had an excess of *A. stenosperma* alleles, while LGs 3, 5, 9, 10, and the inner portion LG 2, an excess of *A. duranensis* alleles. All linkage groups have distorted markers, with LGs 3, 4, 5, and 9 being almost entirely composed of distorted markers. Distorted markers at *P* < 0.05 are indicated by # in Figure 3.

### QTL identification

The framework map, containing 502 markers, was used for QTL analysis. LOD significance threshold estimated for each trait ranged from 2.9 to 22.6, and only QTL with LOD values exceeding these values were included. At least one QTL was detected for 26 of the 29 traits analyzed, with a total of 52 QTL mapped by CIM. No significant QTL was identified for nematode eggs per root_2013 (EGR_2013), 10-seed weight_2009a (10-SW_2009a), and root length (RL). A summary of QTL is provided in Table 2 and described with more details in File S1.

**Table 2:** QTL identified for resistance to *Meloidogyne arenaria* race 1 (RKN), domestication, agronomic and drought-related traits on an *A. duranensis* x *A. stenosperma* F_6_ population

Trait Category	Trait Symbol	LG*^a^*	Position*b*	Nearest Marker/Interval	LOD*c*	Additive Effect*d*	*R*^2^ (%)*^e^*
RKN resistance	GI_2011	2	66.2	seq14F4	6.1	0.957	16.5
		9	40.3	Leg199	6.8	0.868	16.7
	GI_2013	2	68.9	seq14F4/Leg146	6.2	0.908	17.9
		4	74.8	RN12E01	4.2	−0.724	13.4
		9	41.5	Leg199/Leg1Gm	3.4	0.519	8.7
	Log_EGR_2011	2	67.2	seq14F4 / Leg146	15.0	1.053	43.7
		4	39.1	Leg050	3.1	0.432	5.7
		9	41.9	Leg199/Leg1Gm	6.0	0.577	11.9
Drought-related	Log_SCMR_40	2	78.2	TOG896540_664 / Leg069	4.9	−0.332	17.0
		6	88.2	IPAHM-171a / DS_c9222_228	9.3	0.443	31.2
		7	17.4	DS_c4177_150	3.7	0.243	9.5
		10	49.0	SD_c1259p293vAC	3.3	0.228	8.5
	Log_SCMR_60	10	73.5	TOG902928_388 / TOG896385_429	3.1	0.288	11.1
	Log_SCMR_120	4	33.5	gi-832	3.5	−0.326	10.7
		6	84.2	RN0x06	4.4	0.275	13.3
	Log_SLA_40	1	83.6	DS_c1301_572	3.4	0.380	9.9
		9	74.0	SD_c3430p305vAG	3.2	0.353	9.0
	Log_SLA_60	7	82.1	DS_c7479_398	3.3	−0.419	11.1
	Log_SLA_120	4	19.2	TC11B04 / Leg14MGm	3.5	0.476	12.1
		9	3.9	TOG895054_163	6.4	−0.628	20.3
Domestication/	SN_2009a	9	15.0	TOG894454_341	3.7	18.499	13.6
Agronomic traits	SN_2009b	5	85.8	DS_c835_324 / DS_c6779_681	5.9	−65.428	26.0
	Log_SN_2010a	1	33.3	DS_c17991_98	4.8	0.395	15.7
		9	48.3	TOG896362_355 / SD_c2057p484vGT	3.4	0.338	11.4
		10	42.9	IPAHM-689	3.2	−0.290	9.3
	Log_SN_2010b	7	64.5	TOG904989_542	4.1	0.639	18.6
		9	46.3	TOG896362_355 / SD_c2057p484vGT	3.4	0.529	12.9
	Log_10-SW_2009b	3	52.8	DS_c1119_235	3.4	0.238	12.1
	Log_10-SW_2010a	9	44.1	Leg1Gm / TOG896362_355	4.2	0.207	15.2
		10	42.9	IPAHM-689	3.9	−0.170	12.3
	Log_10-SW_2010b	7	64.9	TOG904989_542	3.8	0.191	14.7
	Pod_Constr	1	92.1	TOG904805_259 / RN22A12	3.9	−5.421	14.2
	PL	6	115.0	TOG895571	3.2	−15.379	11.1
	MSH	4	30.6	TC5A07	3.9	2.171	13.4
		5	89.7	DS_c5288_353	4.0	−2.357	10.2
		10	65.8	DS_c10522_129	3.3	−1.803	8.3
	LBL	1	31.1	Leg463_1 / DS_c11080_157	9.9	27.289	32.0
		5	19.5	TOG895690_378	3.7	14.566	8.9
	NLB	8	95.3	TC22C01	3.2	1.025	10.7
		10	40.4	TC31C09 / IPAHM-689	5.1	−1.371	19.1
	ADW	3	0.0	Seq4F10	3.3	3.645	9.3
		6	56.4	Leg346 / TC7C06	5.3	3.602	15.6
		9	63.5	TOG896078_413	3.8	−3.012	10.5
	Log_RDW	5	6.5	TOG896097_531 / Leg231	3.5	0.235	14.3
		6	63.0	TOG896979_290	4.0	0.238	16.4
	Log_RRA	1	33.3	DS_c17991_98	3.5	0.132	11.2
		1	39.0	Ah-193	4.5	0.146	13.9
		10	57.1	TC7H11	3.4	0.160	11.0
	TB	6	56.4	Leg346 / TC7C06	6.7	5.124	21.2
		9	63.5	TOG896078_413	3.1	−3.460	9.5
	PV	4	36.9	TOG906490	3.3	1.793	10.7

LOD, logarithm of the odds; GI, gall index; EGR eggs/g of root; SCMR, SPAD chlorophyll meter reading; SN, seed number; SW, seed weight; Pod_Constr, pod constriction; PL, peg length; MSH, main stem height; LBL, lateral branch length; NLB, number of lateral branches; LBL, lateral branch length; ADW, aerial dry weight; RDW, root dry weight; RRA weight ratio root/aerial part; TB, total biomass; PV, pollen viability.

a Linkage group.

b Map position in Kosambi cM.

c Maximum LOD score.

d Positive values indicate that higher-value alleles come from *A. duranensis* K7988, and negative values indicate that higher-value alleles come from *A. stenosperma* V10309.

e Proportion of the total phenotypic variance explained by the QTL.

#### Nematode resistance:

Three major QTL for both the root-galling (GI) and egg production (EGR) components of nematode resistance evaluated were consistently identified; these mapped in LGs 02, 04 and 09. On LG02, the closest marker was seq14F4 (Ferguson *et al.* 2004), and the QTL mapped in the same marker interval (66.2–68.9 cM), with LOD scores between 6.1 and 15.0, for GI_2011 and 2013, and for EGR_2011. These QTL explained between 16.5 and 43.7% of the total phenotypic variance. For the QTL on LG09, the closest marker was Leg199 (Bertioli *et al.* 2009), in map interval 40.3–41.9 cM, with LOD scores between 3.4 and 6.8, and explaining 8.7–16.7% of phenotypic variance. The third QTL was identified on LG04 for EGR_2011, close to marker Leg050 (Bertioli *et al.* 2009), on map position 39.1 cM, with LOD 3.1,and explaining 5.7% of the phenotypic variance. For all these QTL, resistance was derived from *A. stenosperma* (Table 2 and Figure 3). An additional QTL was identified on LG04 for GI_2013, close to marker RN12E01 (Moretzsohn *et al.* 2005), on map position 74.8 cM, with maximum LOD of 4.2, explaining 13.4% of the phenotypic variance. This was the only QTL that conferred resistance derived from *A. duranensis* (Table 2 and Figure 3).

**Figure 4:**
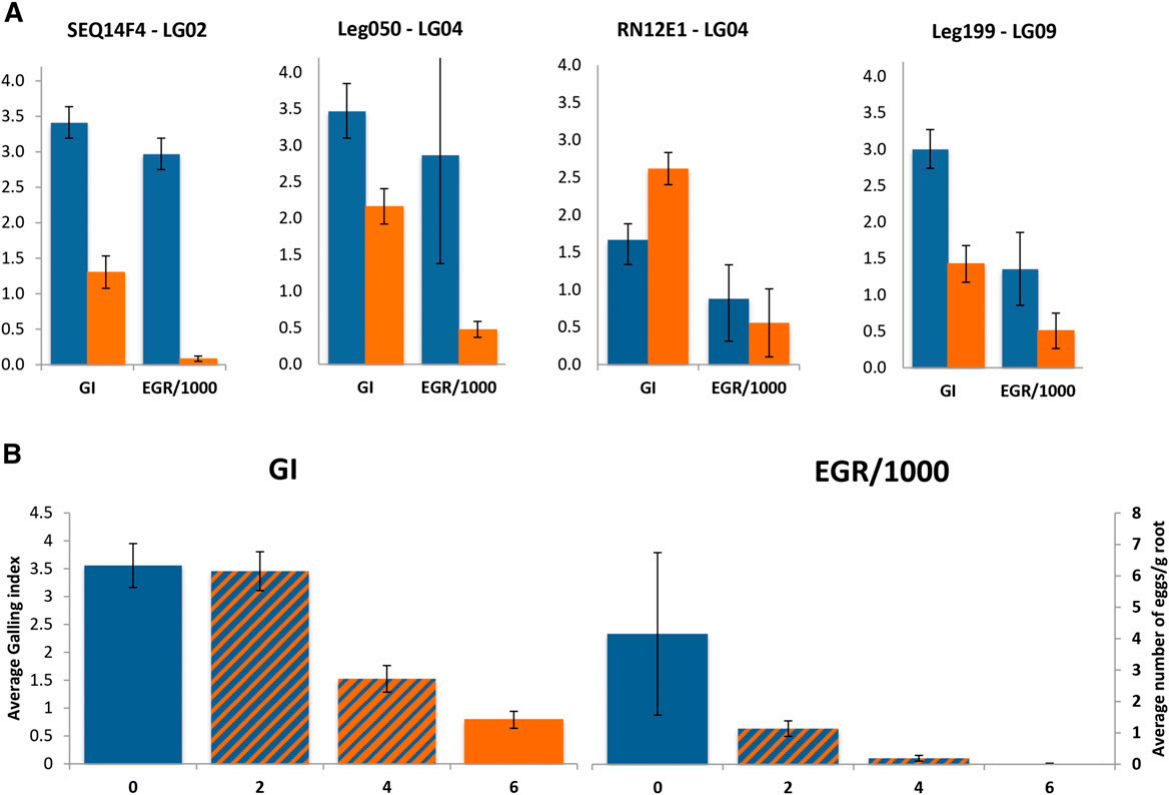
(A) Effect of QTL-linked markers for galling index (GI) and nematode egg production (EGR/1000) on mean phenotypic value (± SE). The markers are distributed on three different linkage groups: Seq14F4 - LG02, Leg050 and RN12E1 – LG04 and Leg199 - LG09. (B) Effect of combination of QTL based on genotypic classes carrying zero to six *A. stenosperma* favorable alleles Seq14F4, Leg050 and Leg199.

With the analyses of the phenotypic effects of nearest markers linked to QTL contributing to nematode resistance, we found that the presence of the *A. stenosperma* allele of locus Seq14F4 (LG02) contributed to a reduction of 61.8% of GI, and 92.6% of EGR (Figure 4A). On average, individuals carrying the *A. stenosperma* allele of locus Leg050 (LG04) had a reduction of 37.7% on GI, and 83.3% on EGR. For the locus Leg199 (LG09), the reductions were 52.5% for GI, and 62.6% for EGR.

#### Drought-related traits:

SLA and SCMR were evaluated at different times of plant development and were treated separately. Five QTL were identified for SLA and seven for SCMR, in seven different LGs. No clear clustering of QTL was observed. The strongest QTL, explaining 31.2% of the phenotypic variation for SCMR_40, was located on LG06, linked to markers IPAHM-171a / DS_c9222_228.

#### Agronomic/domestication traits:

Agronomic and domestication traits were evaluated in different years. A total of 31 QTL was identified. As expected for these polygenic traits, several QTL explaining a small percentage of phenotypic variance were found. Alleles from both parents contributed to an increase of seed number and weight, as well as for MSH, NLB, ADW, and TB. For two domestication traits, pod constriction (PC), and peg length (PL), alleles derived from *A. duranensis* reduced the lengths. For each of these traits, only one QTL was identified. For the other traits (LBL, RDW, RRA, and PV), *A. stenosperma* alleles increased the phenotypic values. A few QTL were consistent between years and some were found in similar positions in different populations. QTL for seed weight (LG07), seed number (LG05), and main stem height (LG04), coincided with the linkage groups with QTL found by (Fonceka *et al.* 2012). Similarly, seed weight (LG07), pod constriction (LG01), and main stem height (LG04 and LG05), were found in similar positions as on the B-population *A. ipaënsis* × *A. magna* (Leal-Bertioli *et al.* 2015b). One QTL for seed number colocalized with a strong QTL for nematode resistance on LG09 (Figure 3).

### KASP primer design and validation on tetraploid backgrounds

**Figure 5:**
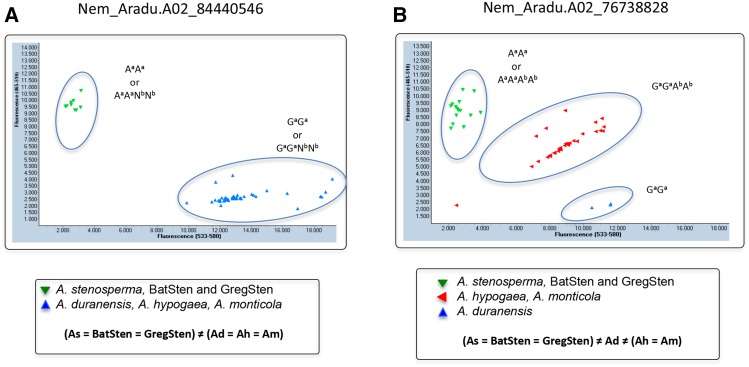
Screenshots of two examples of *Arachis* A-genome SNP genotyping using KASP assays. Both assays (Nem_Aradu.A02_84440546 and Nem_Aradu.A02_76738828) show differentiationcbetween *A. duranensis* V14167 and the A-genome of *A. hypogaea* from the wild species *A. stenosperma* V10309, and the induced allotetraploids BatSten and GregSten. In (A), two clusters are present: one with *A. duranensis*, *A. monticola* and all *A. hypogaea* cultivars, and another with *A. stenosperma* and induced allotetraploids [noted in Table 1 as (As = BatSten = GregSten) ≠ (Ad = Ah = Am)]. In (B), three clusters are present, with an intermediate cluster of *A. hypogaea* and *A. monticola* [noted in Table 1 as (As = BatSten = GregSten) ≠ Ad ≠ (Ah =Am)]. Diploid and tetraploid genotypes are indicated near each cluster. A and G refer to the DNA bases; the subscript letters refer to the A and B subcomponent genomes of peanut. N is used when B subgenome bases are not detected.

Twenty-five KASP assays were designed for the three genomic regions of *A. stenosperma*-derived QTL for nematode resistance, in LG02, 04 and 09. Sixteen successfully distinguished *A. stenosperma* (As) and its derived synthetic allotetraploids from *A. duranensis* (Ad) and the A-subgenome component of all *A. hypogaea* (Ah) tested; seven did not distinguish *A. stenosperma* from *A. duranensis* and *A. hypogaea*, and only two assays failed (Table 1). One assay curiously did not distinguish *A. stenosperma* from the A component of *A. hypogaea*, but did from the As-derived induced allotetraploids. Different useful cluster configurations were observed, and are listed in Table 1: (1) in seven assays, *A. stenosperma* clusters with the induced allotetraploids, BatSten and GregSten, and *A. duranensis* clusters with the peanut cultivars and *A. monticola* [(As = BatSten = GregSten) ≠ (Ad = Ah = Am)] (Figure 5A); (2) In three assays, *A. duranensis* is distinguished from all other genotypes, *A. stenosperma* clusters with the induced allotetraploids, BatSten and GregSten, and *A. monticola* clusters with all peanut cultivars [(As = BatSten = GregSten) ≠ Ad ≠ (Ah = Am)] (Figure 5B); (3) in two assays, *A. stenosperma* is distinguished from all genotypes, and the induced allotetraploids form a cluster, the *A. duranensis*, *A. hypogaea*, and *A. monticola* form a third cluster [(As) ≠ (BatSten = GregSten) ≠ (Ad = Ah = Am)]. Three other clustering configurations can also be useful for distinguishing both induced allotetraploids from peanut: [(As = Ah = Am) ≠ Ad ≠ (BatSten = GregSten)], [(As = GregSten = BatSten = Am) ≠ (Ad = Ah)], and two were useful for distinguishing GregSten: [(As = GregSten) ≠ (BatSten = Ad = Ah =Am)] and (As = Ad = GregSten) ≠ (BatSten = Ah = Am). All useful assays are marked with an asterisk on Table 1. Ten out of the 15 successful KASP assays showed significant Pearson correlation (*P* = 0.05) with nematode resistance (Table 3).

**Table 3:** Pearson correlations between KASP assays and components of nematode resistance on segregating lines of the RIL F6 population *A. duranensis* × *A. stenosperma*. Significant correlations at 0.05 level ≥ I0.344I (values with asterisks)

KASP Assay	LG	Pseudomolecule Position	Trait			
			GI_2011	EGR_2011	Log_EGR_2013	GI_2013
Nem_Aradu.A02_76738828	2	76738828	−0.362*	0.086	−0.358*	−0.497*
Nem_Aradu.A02_84440546	2	84440546	−0.493*	0.084	−0.464*	−0.523*
Nem_Aradu.A02_84440594	2	84440594	−0.555*	−0.325	−0.599*	−0.596*
DS_c1614_886_A02_88903581	2	88903581	−0.693*	−0.505*	−0.750*	−0.722*
TOG894171_695_A02_92486807	2	92486807	−0.600*	−0.021	−0.553*	−0.600*
						
TOG906490_74_A04_106874754	4	106874754	−0.647*	−0.518*	−0.698*	−0.339
Nem_Aradu.A04_109789467	4	109789467	−0.542*	−0.385*	−0.562*	−0.358*
Nem_Aradu.A04_121132127	4	121132127	−0.221	−0.261	−0.291	−0.172
						
Nem_Aradu.A09_112396428	9	112396428	−0.353*	−0.319	−0.312	−0.125
Nem_Aradu.A09_112901114	9	112901114	−0.396*	−0.293	−0.335	−0.142
Nem_Aradu.A09_114001128	9	114001128	−0.400*	−0.207	−0.243	−0.374*
TOG896942_133_A09	9	114770700	−0.197	−0.226	−0.149	0.074
DS_c14276_456_A09_115161052	9	115161052	−0.227	−0.263	−0.229	−0.138
TOG896078_413_A09_116503861	9	116503861	−0.130	−0.235	−0.156	0.011
TOG903757_1119_A09_116533871	9	116533871	−0.130	−0.235	−0.156	0.011

## Discussion

Currently, the only source of resistance to *M. arenaria* used in commercial peanut cultivars comes from *A. cardenasii*. This wild A-genome species harbors a number of loci that reduce RNK infestation (Burow *et al.* 2014) but, to our knowledge, only one, localized on a large chromosomal segment mapping to LG09, has been introgressed into commercial peanut cultivars (Nagy *et al.* 2010). Molecular markers for this chromosomal segment are used in marker-assisted breeding to expedite its incorporation into new cultivars (Chu *et al.* 2011). However, the use of a single source of resistance is clearly vulnerable to being overcome through virulence selection, and there is interest in identifying new sources.

The multiple disease resistances and close relationship of *A. stenosperma* to the A-subgenome of cultivated peanut have stimulated interest in its use in breeding programs. It is now being used in programs in the USA, Brazil, India and Senegal. The *A. stenosperma* accession studied here, V10309, was shown previously to be resistant to *M. arenaria*. The expression of genes involved in the hypersensitive response and production of secondary metabolites related to pathogen defense is triggered shortly following nematode challenge (Proite *et al.* 2007; Guimarães *et al.* 2010; Morgante *et al.* 2013). Microscopically, at least two mechanisms of resistance are apparent: prepenetration (physical or chemical root barriers), and a postpenetration classical hypersensitive response (Proite *et al.* 2008). Here we extend the known resistances of this *A. stenosperma* accession to *M. hapla*, *M. javanica* race 4 and *D. africanus* (Figure 1), and genetically map the resistance for *M. arenaria*. For mapping, we worked in the genetically simplified context of a diploid population. The maternal parent of this population was the most probable A-genome ancestral species of cultivated peanut *A. duranensis* (accession K7988), and the paternal *A. stenosperma*. While the close relationships of these species to the A-subgenome of *A. hypogaea* ensure a good chance that QTL will be applicable for crop breeding, the diploid genetics reduces allelic interactions and avoids complexities of tetrasomic recombination (Leal-Bertioli *et al.* 2015a).

Although the main focus of this work was the identification of QTL for nematode resistance, this population was also evaluated for several other traits. The parents of the mapping population had similar values for drought-related traits (SCMR and SLA); nevertheless, transgressive segregation was observed, and QTL and marker associations were identified. Also, although *A. stenosperma* and *A. duranensis* are both wild species, they differ somewhat in phenotypes that are strongly selected during domestication: *A. duranensis* has shorter pegs and pod constrictions. Transgressive segregation was also observed for these traits, with 31 lines having shorter pegs and pod constrictions than both parents. QTL were identified for these and other plant architectural traits. Many of these traits are complex and quantitative, and will depend on environment and genetic ploidy (Leal-Bertioli *et al.* 2012). Nevertheless their identification enriches the information content of this A-genome map, and they can be easily cross-referenced to the genome sequence of *A. duranensis*.

Four QTL that contribute to RKN resistance were identified, on LG02, 04 and 09. For three of them (closest linked markers Seq14F4, Leg050 and Leg199), the presence of the *A. stenosperma* alleles greatly reduces both root-galling (GI) and egg production (EGR/1000) (Figure 4, A and B). For the other QTL, with closest linked marker RN12E01, the effect was opposite: *A. stenosperma* alleles increased root-galling. It is worth noting that, for the diploid population, the susceptible parent (*A. duranensis* K7988) is much more resistant than *A. hypogaea*. Therefore, the effects of the resistances conferred by wild species alleles in the context of the highly susceptible cultivated peanut genetic background are likely to be larger than the effects measured here. The genome location of all these QTL is different to the *A. cardenasii* chromosomal segment currently used in commercial cultivars. Therefore, in principle, multiple sources of resistance, derived from different QTL (Figure 4B) could be harbored in peanut cultivars for improved, and potentially more durable, resistance.

To deploy these resistance QTL for crop improvement, we have previously developed *A. stenosperma*-derived artificially induced allotetraploids that are sexually compatible with cultivated peanut (Leal-Bertioli *et al.* 2015c). In this study we developed new KASP markers around the QTL of interest using the genome sequence of *A. duranensis* (http://www.peanutbase.org); confirmed the marker associations with nematode resistance and tested them in tetraploid genotypes. *A. stenosperma*-derived induced allotetraploids (BatSten and GregSten) were distinguished from all the peanut cultivars, including Tifguard, which harbors *A. cardenasii*-derived RKN resistance. In ongoing work these markers will be used to facilitate the selection of backcrossed progeny that harbor the *A. stenosperma* QTL of interest, and the testing of their function in a tetraploid genetic background.

## Supplementary Material

Supporting Information
