# Supplementary material for: Genetic Mapping of Resistance to Meloidogyne arenaria in Arachis stenosperma: A New Source of Nematode Resistance for Peanut
Source: G3 (Bethesda). 2015 Dec 10;6(2):377–90. doi: 10.1534/g3.115.023044 (PMC4751557; doi:10.1534/g3.115.023044)
Supplement: Supporting Information [file supp_6_2_377__index.html]

Genetic Mapping of Resistance to Meloidogyne arenaria in Arachis stenosperma: A New Source of Nematode Resistance for Peanut — Supporting Information 

# Genetic Mapping of Resistance to *Meloidogyne arenaria* in *Arachis stenosperma*: A New Source of Nematode Resistance for Peanut

## Supporting Information for Leal-Bertioli *et al.*, 2016

**Files in this Data Supplement:**

- File S1 - This Excel file contains all the map and marker information for this manuscript. The information is organized in three tabs: *Framework map*: Marker names, Linkage Group, Map position, marker type, sequence, literature reference, primer sequences, GenBank reference and genotyping information. This map was produced using MapMaker and was used to identify QTLs. *Traits*: Information of the traits evaluated: category, trait name, abbreviation and values for each genotype. *QTLs*: Information about the QTLs identified. (.xlsx, 455 KB)
